# Bioassay Development for Bispecific Antibodies—Challenges and Opportunities

**DOI:** 10.3390/ijms22105350

**Published:** 2021-05-19

**Authors:** Ames C. Register, Somayeh S. Tarighat, Ho Young Lee

**Affiliations:** 1 Biological Technologies, Department of Analytical Development and Quality Control, Genentech—A Member of the Roche Group, 1 DNA Way, South San Francisco, CA 94080, USA; register.ames@gene.com; 2 Cell Therapy Analytical Development, Department of Cell Therapy Engineering and Development, Genentech—A Member of the Roche Group, South San Francisco, CA 94080, USA; tarighat.somayeh@gene.com

**Keywords:** bispecific antibodies, bioassays, mechanisms of action, binding assays, potency assays

## Abstract

Antibody therapeutics are expanding with promising clinical outcomes, and diverse formats of antibodies are further developed and available for patients of the most challenging disease areas. Bispecific antibodies (BsAbs) have several significant advantages over monospecific antibodies by engaging two antigen targets. Due to the complicated mechanism of action, diverse structural variations, and dual-target binding, developing bioassays and other types of assays to characterize BsAbs is challenging. Developing bioassays for BsAbs requires a good understanding of the mechanism of action of the molecule, principles and applications of different bioanalytical methods, and phase-appropriate considerations per regulatory guidelines. Here, we review recent advances and case studies to provide strategies and insights for bioassay development for different types of bispecific molecules.

## 1. Introduction

The concept of the bispecific antibody (BsAb) has been around for more than 50 years, but within the last 20 years, activity and interest in the field of study has skyrocketed [1,2]. Publications describing hundreds of BsAbs can be found in the scientific literature, and more than 100 BsAb clinical candidates are currently under development [3,4]. A handful of BsAbs have obtained health authority approval for use and are currently marketed as therapeutics in a number of disease areas (e.g., blinatumomab, emicizumab) around the world, highlighting the therapeutic potential of engaging two targets within a single molecule [4]. This is attributed to advanced biotechnologies, enhanced manufacturing knowledge of therapeutic antibody products, and strong scientific rationale for the development of biologics with the ability to engage more than one target [5,6].

BsAbs are typically designed to possess the epitope specificity and manufacturability of a conventional monoclonal antibody (mAb) but are engineered to bind two distinct targets instead of one. The actual structure of a BsAb can vary widely, and depends on a number of factors including the intended mechanism of action (MoA) of the BsAb and desired pharmacokinetic/pharmacodynamic (PK/PD) properties [7,8]. Development and commercialization of BsAbs, to engage multiple targets using only one therapeutic, has gained significant attention recently, shifting industry focus and investments on this effective therapeutic strategy.

In this review, we discuss challenges and opportunities associated with developing bioassays for BsAbs with a particular focus on recent advances in bioanalytical approaches, as supported by multiple case studies.

### 1.1. Diverse Formats of BsAb

**Figure 1:**
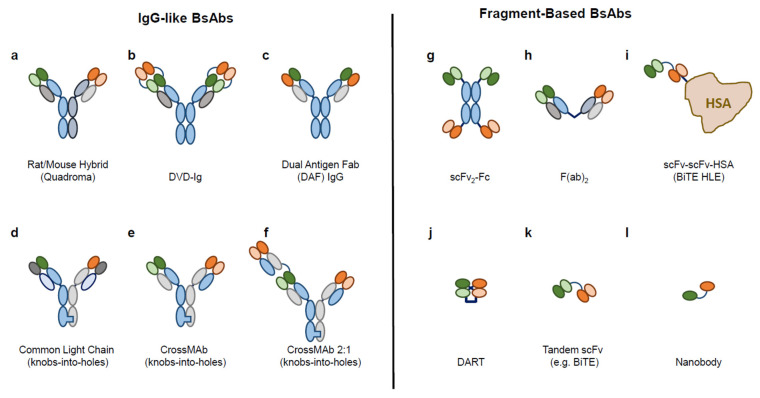
Examples of BsAb formats and structural diversity: (**a**–**f**) IgG-like BsAbs and (**g**–**l**) fragment-based BsAbs.

There are more than 100 distinct BsAb formats described and reviewed in the literature, but they generally fall into two categories: IgG-like and fragment-based (see Figure 1 and Wang et al. [9]).

DVD-Ig: dual variable domain immunoglobulin; scFv: single-chain variable fragment; Fab: antigen-binding fragment; HSA: human serum albumin; BiTE: bispecific T-cell engager; HLE: half-life extended; DART: dual-affinity re-targeting antibody.

The IgG-like BsAbs approximate the structure of a traditional mAb and typically contain an Fc domain and two antigen binding domains. However, many designs incorporate multiple copies of one or more antigen binding domains, allowing for avidity binding of one or more targets (Figure 1a–f; [10]). For example, an IgG-like anti-human epidermal growth factor receptor 2 (aHer2)/aCD3 bispecific molecule was engineered to include two low-affinity Her2 binding domains, thereby increasing the selectivity of the BsAb for cells overexpressing Her2 and increasing selective killing of tumor cells over Her2-expressing bystander cells [11]. IgG-like BsAbs tend to have longer serum half-lives due to the presence of an Fc domain that can interact with neonatal Fc Receptor (FcRn), and they can be easily engineered to either maximize or minimize interactions with FcgammaRs, allowing for flexibility in regards to effector function activity such as antibody-dependent cellular cytolysis (ADCC), antibody-dependent cellular phagocytosis (ADCP), and complement-dependent cytotoxicity (CDC) as desired [12]. IgG-like BsAbs can be challenging to manufacture, as many platforms require in-vitro or in-vivo assembly of two distinct half antibody pairs, resulting in product-related impurities stemming from chain mispairing events that can be difficult to separate from the desired product [9]. However, a number of technologies have been developed to overcome these challenges and maximize BsAb formation including knobs-into-holes, Cross mAb, and common light chain, among others [13,14,15,16].

In contrast, fragment-based BsAbs are typically much simpler to manufacture, as they are smaller and less structurally complex. Many fragment-based BsAbs are made by combining scFv fragments of different specificities (see Figure 1g–l), and they often self assemble from a single polypeptide chain (no opportunity for chain mispairing) [17]. Their small size can lead to better tissue penetration, and it has been postulated that their small size and conformational flexibility enable a more potent receptor activation, for example when bridging two cell types, compared to their larger counterparts [7,10]. However, they tend to have very short serum half lives due to the lack of an Fc domain. For example, while efficacious, blinatumomab treatment requires continuous infusion due to its extremely short serum half life (~2h; [18]). Several fragment-based structures have been developed to increase serum half, including appending scFv fragments to Fc domains or Human Serum Albumin (HSA) [10,19]. As with IgG-like BsAbs, there is a wide range of structural variability and avidity of binding available with this class of BsAb molecules.

### 1.2. Mechanisms of Action of BsAb

**Figure 2:**
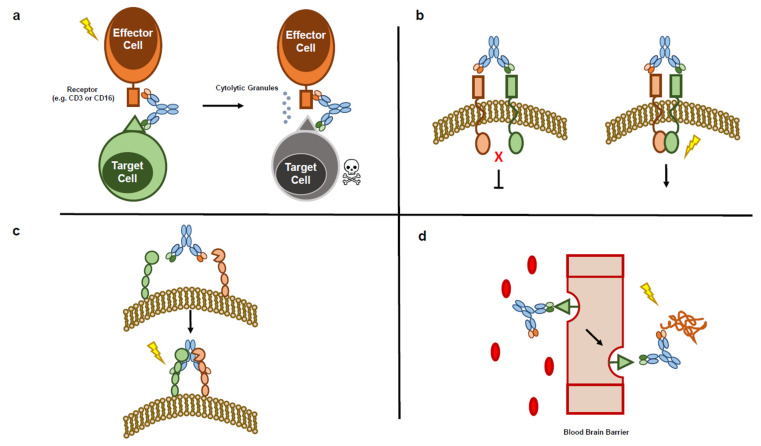
Mechanisms of actions of BsAb: (**a**) Schematic diagram of cell-bridging BsAb MoA (e.g., TDB or NK-recruiting BsAb); (**b**) Schematic diagram of receptor activating/inhibiting MoA (e.g., receptor dimerization inhibitor or activator); (**c**) Schematic diagram of cofactor mimicking MoA (e.g., emicizumab); and (**d**) Schematic diagram of “homing” BsAb MoA (e.g., blood brain barrier crosser).

**Figure 3:**
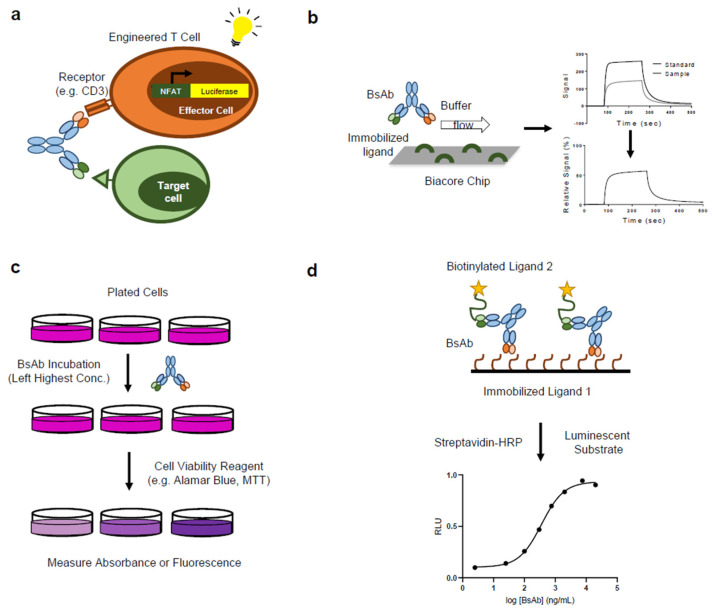
Representative bioassays for BsAb: (**a**) Reporter gene T-cell activation assay; (**b**) Single-arm binding SPR assay; (**c**) Cell proliferation assay; (**d**) Bridging ELISA. MTT:3-(4,5-dimethylthiazol-2-yl)-2,5-diphenyl-2H-tetrazolium bromide.

Due largely to the high level of interest in BsAbs as potential therapeutics and because of their structural diversity in design, both the scientific literature and clinical development pipeline contain numerous examples of BsAbs whose MoAs span a wide range [3]. For the purpose of this review, we will sort the BsAbs into four general classes: cell-bridging BsAbs, receptor/ligand blockers or activators, cofactor mimetics, and “homing” BsAbs (Figure 2; [3,20]).

#### 1.2.1. MoA Type 1—Cell-bridging BsAbs

Cell-bridging BsAbs bind two distinct cell surface receptors—one on the surface of an effector cell and one on the surface of a target/tumor cell—resulting in activation of downstream signaling networks and killing of the target cell. One of the most prevalent examples of this MoA currently under clinical development is the T-cell dependent BsAbs (TDBs; [1]). These molecules most often target CD3e within the T-cell receptor (TCR) of cytolytic T cells and a tumor-specific antigen on the surface of target cells [8,21,22,23,24,25,26,27,28,29,30,31,32,33,34]. However, there are examples of BsAbs that activate T cells by engaging other epitopes, such as CD5 or co-stimulatory receptors such as CD28 [35,36]. Bridging of the target cell and the T cell by the BsAb leads to the formation of an immunological synapse, inducing T-cell activation and resulting in the release of perforin and granzymes that lyse the target cell [37]. Thus, TDBs harness a patient’s own immune system to kill tumor cells independent of TCR epitope specificity by circumventing activation through the major histocompatability complex [2]. TDB immunotherapy is similar in concept to CAR-T therapy, in which a patient’s T cells are extracted and engineered with a chimeric antigen receptor (CAR) designed to recognize and kill tumor cells [38]. However, while TDBs are often more complex and difficult to produce than a standard mAb biologic, they are currently cheaper and less logistically challenging to manufacture than CAR-T therapies, which must be prepared individually for each patient [39]. Additionally, TDBs can have more favorable safety profiles compared to CAR-T therapies, with fewer and less severe adverse events such as systemic cytokine release syndrome—the most common adverse event associated with immune-modulating therapies [27,40,41]. In addition to TDBs, there are several examples of BsAbs that recruit and activate NK cells by simultaneously binding CD16 (FcgammaRIII) and a tumor-specific receptor [42,43,44,45], as well as a BsAb that recruits and activates macrophages by targeting CD89 [46].

#### 1.2.2. MoA Type 2—Receptor/Ligand-Blocking or -Activating BsAbs

By virtue of their ability to target more than one receptor, BsAbs can be developed to target and activate a receptor in a specific cellular context (e.g., a therapeutically-relevant complex). This allows for a level of selectivity that cannot be achieved with conventional mAbs alone or in combination. For example, the anti-Fibroblast Growth Factor Receptor (aFGFRI)/anti-β-Klotho (aKLB) BsAb activates the FGFRI/KLB receptor complex, leading to weight loss and a reduction in obesity-linked disorders in preclinical models [47]. By selectively targeting FGFRI/KLB, the molecule activates FGFRI when complexed with KLB, thereby avoiding widespread FGFRI activation—FGFRI receptor is expressed in a wide range of tissues—and reducing the unintended side effects associated with mAb FGFRI agonists.

In addition to acting as receptor agonists, BsAbs can also be effective receptor antagonists. Resistance to various Her2-targeting mAbs (e.g., trastuzumab) has led to the development of novel therapeutics for blocking Her2-associated signaling, including several BsAbs [48]. While many of these molecules are TDBs (MoA discussed above), there are also examples of BsAbs that bind to Her2 and Her3, preventing ligand-activated Her3 from heterodimerizing with Her2, and dampening PI3K signaling in Her2-overexpressing cancers [48]. There is also an example of an antibody-drug conjugate (ADC) BsAb that targets two distinct, non-overlapping epitopes on Her2, leading to more efficient internalization, lysosomal degradation, and release of cytotoxic payload [49]. Beyond treatments for Her2-overexpressing cancers, there are many examples of BsAbs that target combinations of receptors and/or cognate ligands, as well as cytokines [50,51,52,53,54,55,56,57,58,59]. These BsAbs sometimes serve the same purpose as that of a combination treatment of mAb therapeutics, but there are instances in which a BsAb provides a particular advantage. For example, an aCTLA4/PD1 BsAb was developed to preferentially inhibit CTLA-4 on PD1+ cells, leading to fewer adverse events associated with immune activation than have been observed when treating patients with combinations of the conventional mAb aCTLA-4 and aPD1/L checkpoint inhibitors [60]. Monovalent targeting of CTLA-4 significantly reduces the ability of the BsAb to inhibit CTLA-4, but monovalent binding has a much lower impact on the ability of the molecule to inhibit PD1 compared to a conventional bivalent aPD1 mAb. As a result, the BsAb is able to saturate CTLA-4 receptors on PD1+ cells, without widespread inhibition of CTLA-4 leading to fewer adverse events. Bispecific targeting of CTLA-4 and PD1 with this BsAb also leads to internalization and degradation of PD1—an effect that is not observed with combinations of aCTLA-4 and aPD1 mAbs.

#### 1.2.3. MoA Type 3—Cofactor Mimicking BsAbs

Emicizumab (marketed name Hemlibra^®®^) is a BsAb that was developed to treat hemophilia A. The BsAb binds to coagulation Factors X and IX and is therefore able to play the role of Factor XIII—the coagulation factor missing in many hemophilia A patients [61,62].

#### 1.2.4. MoA Type 4—“Homing” BsAbs

For the purposes of this review, “homing” BsAbs are molecules in which one arm serves to deliver the molecule to a specific, often hard-to-reach location. There are multiple examples with a diverse range of therapeutic targets. Several BsAbs have been developed that are able to cross the blood-brain barrier by targeting the transferase receptor [63,64,65]. Once across the barrier, the non-transferase receptor arm can target the therapeutic target of interest (typically amyloid beta-protein and other Alzheimer’s Disease targets). Additionally, there is an example of a tandem scFv BsAb that targets activated platelets and sca-1, helping to bring stem cells to the location of injury; it is being explored for the treatment of myocardial infarction [66]. An aCD63/aHER2-ADC has been developed, in which binding to CD64 targets the molecule to the lysosome while the aHer2 portion provides tumor specificity, leading to a more efficient release of the conjugated drug [67]. There is also an example of a BsAb with one epitope designed to gain entry into the late endosome, where it is able to neutralize Ebola virus [68].

### 1.3. Challenges and Opportunities of BsAb Bioassay Development

Concurrent to the development of these complex biological products with multiple modalities is the need to develop bioassays that are not only accurate and reproducible, but also adequately reflective of the proposed mechanism(s) of action. Well-developed bioassays are critical to the characterization and control of biological products, as well as to the interpretation of clinical study results. BsAb bioassay development presents a unique set of challenges for assay design, such as the ability to fulfill the desired performance of the assay (i.e., to capture the dual activities and potential synergistic effects of the molecule) preferably using a single assay format, and to detect multifaceted structural changes [69]. Depending on the molecule’s MoA, several bioassays might be necessary for characterization in addition to a main potency assay in the control system. For example, cell-killing, cytokine secretion, receptor internalization [70], effector function (ADCC, ADCP), and surface marker expression assays might need to be developed for the characterization of bispecific molecules for later stages of product development in addition to the one most MoA-relevant bioassay selected and validated for release, stability, and comparability testing for product licensure. A number of technologies were developed to overcome these challenges to characterize BsAb, and selected case studies are described in the Section 3.

## 2. Strategies and Considerations for BsAb Bioassay Development

### 2.1. Phase-Appropriate Approach

A phased approach to the development and implementation of bioassays for biotherapeutics is widely accepted by industry and regulatory agencies, and the similar principles apply to bispecific therapeutics. It is often advantageous and preferred to start with a binding method for the early phases of product development. Most commonly implemented binding assays include enzyme-linked immunosorbent assays (ELISAs) or surface-plasmon resonance (SPR) technologies. More complex and MoA-reflective cell-based bioassays are developed by later phases, and they are validated before marketing application submission. However, it is recommended that a relevant MoA-based bioassay is developed earlier, not only to gain a greater process and product understanding but also to gain a better understanding of the method’s performance prior to pivotal clinical trials. Cell-based bioassays should be qualified and monitored over the span of the clinical development to have a true understanding of the critical steps and components of the assay in most cases. The selection of the bioassay should be driven by the product’s therapeutic MoA. In cases where the MoA is simply binding to a target, a surrogate method, such as a protein binding or competitive binding assay, may be sufficient to determine potency. Developing robust and quality-control (QC)-suitable cell-based bioassays is more challenging than developing non-cell based binding assays [71,72]. There are case studies of implementing surrogate, non-cell-based bioassays in the commercial control system if the surrogate assay has demonstrated a good correlation in a bridging study using degraded product and other samples with the MoA-reflective cell-based assay.

### 2.2. Mechanism of Action

Design strategies for bioassays are driven by the drug’s intended physiological MoA. Unlike other analytical techniques, bioassays are almost always unique for each therapeutic. A well-designed bioassay will accurately capture the biological activity of a drug candidate. As shown in Figure 2, common MoAs of bispecific therapeutics include direct binding to soluble targets (e.g., ligands, cytokines and enzymes), or to cell-surface receptors in either an inhibitory or agonistic manner.

Each MoA will require a different approach when considering the bioassay design. In the case of BsAbs and related recombinant proteins, secondary, tertiary, or synergistic MoAs may be discovered during development. This biological complexity further contributes to the challenge of developing MoA-reflective assays to capture the candidate molecule’s putative therapeutic biological activity [50,73,74,75,76]. In some cases where multiple MoAs exist in a single molecule, a combination assay that measures all MoAs in a single assay may be suitable for product release and stability testing, with secondary characterization assays developed to measure the individual activities of each MoA if applicable. Otherwise, multiple bioassays would be necessary to fully characterize the molecule’s activity. It is not required to have all bioassays for release, instead only the assay with the most MoA-relevant and stability indicating bioassay can be selected as the release potency assay while the other assays are used for characterization.

### 2.3. Overall BsAb Characterization Strategy

Efficacy and safety assessments of BsAbs rely on the successful development of a pharmacologically and clinically relevant bioanalytical strategy that most importantly can reflect the biological activities of these dual-targeting antibodies and can differentiate higher order structure, potency, and efficacy.

It is most vital to develop characterization and bioanalytical approaches to study important quality attributes [77] including overall stability, fragmentation/aggregation/immunogenicity, antigen specificity, affinity, on and off rates, avidity (for molecules with two targets on the same cell), and MoA/biological activity.

While BsAbs require bioassays to measure two binding events, the choice of the appropriate bioassay will also depend on the assay format, assay platform, critical reagents, and, importantly, the BsAb target profile. Following the successful development of the pharmacologically relevant BsAb format, the analytical strategy is outlined to first characterize the independent or simultaneous binding affinities and the preferential binding of BsAb to their dual-antigen targets. Widely used bi-functional quantitative assay formats to enable target-specific capture and detection of binding properties include flow cytometry and ligand-binding immunoassay setups. A range of other assay platforms (ELISA, SPR, ADCC, competitive flow cytometry, etc.), whose selection relies on BsAb format, MoA, and target profile, are used to address bioanalytical questions for BsAbs. These assays are listed in Table 1 and further discussed in Section 3.

Meaningful bioanalytical approaches are also needed for immunogenicity and PK/PD assessments to determine the safety and efficacy of BsAbs [78,79]. Immunogenicity is defined as the unwanted immune response of the host against the therapeutic BsAb. In addition to altering the PK of a target through changing its clearance, immunogenicity is responsible for infusion-related reactions and in some cases, reduced treatment efficacy [80]. Immunogenicity is clinically assessed by the detection of anti-drug antibodies, consisting of IgM, IgG, IgE, and/or IgA isotypes [81]. The bioassays employed to assess immunogenicity include binding immunoassays such as ELISA to detect all isotypes capable of binding the therapeutic BsAbs, and neutralization assays (in-vitro cell-based assays or competitive ligand-binding assays) directed at the biologically active site, to inhibit the functional activity of BsAb. Major histocompatibility complex-II (MHC-II)-Associated Peptide Proteomics (MAPPs) assay can screen and quantitate naturally processed and presented MHC-II peptides on the surface of antigen-presenting cells, which are then further characterized for immunogenicity using in-vitro assays. T-cell epitope-mapping prediction tools are also used to identify the CD4 T cell epitope within the amino acid sequence of the therapeutic antibody and determine the strength of peptide binding to HLA molecules [82,83]. PK for biologics is often at least partially determined by FcRn-mediated recycling. In-vitro assays designed to measure binding of a therapeutic antibody to FcRn via its Fc domain, including SPR-based FcRn binding assays and FcRn-affinity chromatography, have been shown to be indicative of FcRn-mediated clearance and are frequently used to assess potential impacts to PK [84,85].

## 3. Bioassays for Bispecific Antibodies and Case Studies

### 3.1. Bioassays for Biotherapeutics

For biotherapeutics, a selective, physiologically relevant bioassay is essential to report on the product’s potency and stability, by providing an assessment of the molecule’s biological activity. Bioassays, in principle, can range from recognition of a particular antigen in a simple binding method, through systems as complex as blocking an inhibitory ligand that restores a co-stimulatory effect in a cell-based method. Selection of an appropriate method has its challenges rooted not only in the need to mimic the MoA, but also because bioassays can be costly to develop, perform, transfer, and maintain. Despite efforts to implement measures to ensure method control, cell-based bioassays can be inherently variable and often lack the precision and robustness of biophysical methods simply because they use living organisms, tissues, or cells.

While the general principles of bioassay design and strategy (e.g., measuring antigen target binding and biological activities) apply to bispecific antibodies, developing bioassays for bispecific antibodies requires unique considerations as bispecific antibodies bind two different targets with distinct mechanisms of action from monospecific biotherapeutics. A diverse range of bioanalytical assays have been developed and employed to study BsAbs, including methods designed to assess binding, potency, biological function, and purity. Figure 3 depicts a few of the methods involved in the various types of BsAb bioassays, which are further discussed in the following sections, and case studies are summarized in Table 2.

### 3.2. BsAb Bioassay: Binding Assays

ELISA and SPR are commonly used for in-vitro characterization of antigen binding for BsAbs. ELISAs are advantageous in that they are sensitive, typically fast to develop compared to cell-based assays, relatively inexpensive, and can be performed in complex matrices (e.g., cell lysates) [98,99]. Bridging ELISAs and competitive binding ELISAs can also provide information on the ability of the BsAbs to bind both antigens simultaneously, and they can therefore potentially be used as MoA-reflective potency assays, at least during initial development phases. The case studies presented below provide examples of competitive and bridging ELISAs used to confirm the simultaneous binding of two different targets for a tetravalent IgG-like BsAb. Despite their advantages, ELISA assays have drawbacks, one of which is that they are end-point assays and do not provide information on binding kinetics such as on and off rates [100]. In contrast, SPR measures continuous binding in a flow cell without the need for chemical labels, and the entire binding event can be analyzed in real time (association and dissociation). This allows for the determination of both kinetic and thermodynamic parameters through various data analyses [101]. SPR assays can also be designed to measure binding to two targets simultaneously, and are also potential candidates for MoA-reflective potency by binding assays. The case studies presented below provide two examples of assay formats for the purpose of measuring concurrent antigen binding by SPR.

Drawbacks for both ELISA and SPR are that it can be difficult to measure the impact of either target density (avidity effects) on BsAb binding, which can be important factors for a BsAb’s activity in an in-vivo context. SPR allows for control of target density by depositing different amounts of capture ligand on the sensor chip. Binding of the therapeutic antibody can then be characterized under the different conditions to investigate the effects of receptor density on binding affinity/avidity [102]. However, the fact that BsAbs bind two targets, with varying levels of avidity depending on structure/format, can make this type of experiment challenging to design and interpret using label-free mass-based detection by SPR [103]. Additionally, there are open questions with respect to the in-vivo relevance of binding events [e.g., how relevant is binding to an immobilized ligand on a chip, or is solution-based association of a truncated ligand (such as a peptide or extracellular domain) reflective of binding to a cell surface receptor?] [104]. Investigators have used SPR to measure binding of aCD20 mAbs to membrane bound CD20, and developed sophisticated software that makes it possible to extract and analyze individual binding events from heterogeneous mixtures. There have also been reports of affixing multiple ligands on a sensor surface in a solution-like context using DNA-directed immobilization using SPR, which would be useful for characterizing BsAbs. New biosensor technologies that allow for discrete detection of both binding events and precise control over surface density, as well as advances in SPR data analysis and experimental design, may provide avenues for more thoroughly investigating complex binding events under increasingly biologically relevant conditions in the future [105,106,107,108,109].

- Case Study: ELISA to detect simultaneous target binding by a tetravalent BsAb [86]: The authors developed a tetravalent BsAb composed of two different variable heavy (VH) domains on an IgG framework (tetra-VH IgG). The structure was meant to be an alternative to dual-action Fab (DAF) molecules, which are often difficult to generate via mutagenesis and additionally may not be able to bind both targets simultaneously, due to overlapping binding surfaces. Three bispecific tetra-VH IgGs were created (CD40/OX40, 4-1BB/CD40, OX40/4-1BB). In order to confirm that the BsAbs were able to bind both targets simultaneously, a competition ELISA assay was developed. The BsAb was immobilized on a plate and incubated with non-biotinylated ligand (for example OX40), followed by biotinylated ligand (for example OX40 or CD40). For each BsAb, binding of the biotinylated ligand was only inhibited by binding of the biotinylated ligand of the same specificity, while the ligand of the other specificity retained binding, suggesting that the BsAb is able to bind both targets simultaneously. To confirm that each arm of the BsAb (VH_OX40_-VH_4-1BB_ Fab) can simultaneously bind each target, a bridging ELISA assay was used. An assay plate was coated with OX40 and allowed to bind to the VH_OX40_-VH_41BB_ Fab before being incubated with varying concentrations of biotinylated 4-1BB or biotinylated OX40. Dose-dependent binding of 4-1BB was observed, while no binding of OX40 was observed, supporting the conclusion that each arm of the BsAb is able to simultaneously bind both targets.
- Case Study: SPR to measure kinetics and binding affinity of Ang-2/VEGF BsAb [88,89]: The authors evaluated a humanized bivalent-BsAb generated for the neutralization of angiopoietin-2 (Ang-2) and vascular endothelial growth factor A (VEGF-A) [88]. Using SPR technology to characterize the kinetics and affinity of binding, the authors developed an assay to cover two binding events simultaneously, which can be reported as one response. Assay setup utilized a Biacore™ instrument and commonly used CM5 chips, and followed a scheme of sequential additions of the CrossMab and then Ang-2 to immobilized VEGF. The second binding event (Ang-2 binding to the VEGF-bound CrossMab) included surface regeneration. As a result, Ang-2-binding response is dependent on the amount of VEGF-bound CrossMab molecules and therefore reflects the actual bridging signal. In this assay, SPR-detected bridging signal reflects the active concentration of Ang-2- and VEGF-binding molecules, where the loss of overall binding can be attributed to either the VEGF or the Ang-2 binding contribution, and therefore, covers both antigen interactions. A modified SPR-based dual-binding assay was developed by Meschendoerfer et al. [89] to address the pitfalls associated with the bridging assay—specifically the change of antigen activity upon immobilization. The main objective was to allow for the individual assessment of both targets in solution while avoiding the need for immobilization and regeneration of the target. They determined the individual VEGFA-121 and Ang2 activities of an anti-VEGFA-121/Ang2 BsAb where an anti-human-Fab capture system (for the Ang2 antigen) was used to measure different antibody concentrations with the same Biosensor (regeneration cycles included). The findings suggested that comparable binding signals can be read from individual injections, when compared to an approach with sequential antigen injection. Using this assay, they showed that simultaneous binding can be calculated based on both individual readouts: two binding events can be measured, and the third parameter can be accurately calculated based on these measurements.

#### Cell Surface Ligand Binding Assays

Binding properties of investigational BsAbs to their targets can also be assessed by flow cytometry, which can be used to measure binding specificity and selectivity of BsAbs in a cellular context—information that is not captured in a traditional SPR or ELISA-based binding assay. Flow cytometry, is a fluidics and optics-based method that evaluates fluorescently-labeled cell suspensions in a single cell flow to capture receptor- or antigen-binding events in intact cells. However, flow cytometric analysis of antibody binding is an indirect measurement of kinetic values and it should be used in combination with SPR analysis to provide truly comprehensive, label-free, and accurate kinetic data for the antibodies being studied.

In addition to flow-cytometry assays, cell-based reporter assays have been developed to measure gene expression in response to disruption of an inhibitory binding interaction, such as PD-L1/PD-1 [93]. As a result, these assays provide a functional measure of BsAb binding as opposed to, for example, directly measuring binding affinity by SPR. In the case studies discussed below, variations of flow cytometric analysis are used to demonstrate the preferential binding, receptor blocking, and avidity of binding to dual-antigen-expressing target cells. In addition, application of a reporter assay to assess BsAb-mediated blockade of receptor-ligand interaction between the antigen-expressing tumor cells and effector cells is reviewed.

- Case Study: Flow cytometry-based binding and blocking assays for GPC3/CD47 BsAb [92]: To develop a potential immune-modulating therapeutic to treat hepatocellular carcinoma (HCC), the authors designed a novel BsAb directed against the HCC-associated antigen Glypican-3 (GPC3) and CD47, an inhibitory innate immune checkpoint that inhibits ADCP by binding to SIRPa on myeloid cells. Due to the fact that CD47 is widely expressed on both healthy and cancerous cells, treatment with anti-CD47 mAbs is associated with toxicity. Therefore, the authors sought to direct the ADCP-enhancing activity of targeting CD47 to GPC3+ tumor cells using a bispecific approach. Several flow cytometry-based binding assays were used to demonstrate selective targeting of GPC3+ cells. For example, wild-type Raji cells (GPC3-) were labeled with a fluorescent dye and mixed in a 1:1 ratio with unlabeled Raji cells engineered to express GPC3 (Raji-GPC3^H^) prior to incubation with GPC3/CD47 BsAb or anti-CD47 mAb. Following incubation with a FITC-labeled secondary antibody, labeled and unlabeled cells were separated by flow cytometry and the binding of the BsAb to each cell population was assessed. The results showed higher levels of BsAb binding to Raji-GPC3^H^ cells compared to the wild-type cells. In contrast, no difference in binding was observed for an anti-CD47 mAb. The authors further tested the ability of the BsAb to block the interaction between CD47 and SIRPa in each cell type using a competitive flow cytometry assay. Wild-type and Raji-GPC3^H^ cells were incubated with biotinylated SIRPa-mF in the presence of anti-CD47 mAb or GPC3/CD47 BsAb, followed by the addition of FITC-labeled streptavidin. The results showed that the BsAb prevented SIRPa binding more effectively in the Raji-GPC3^H^ cells, while the anti-CD47 mAb showed similar blocking activity in both cell types. The results of the flow cytometry-based binding assays demonstrate preferential binding and blocking activities of the GPC3/CD47 BsAb in GPC3+/CD47+ compared to GPC3-/CD47+ cells in vitro. These results suggest that the bispecific targeting of GPC3+ and CD47 may preferentially induce killing of GPC3+ tumor cells by ADCP.
- Case Study: Flow cytometry-based assay to characterize binding activity of PD-L1xCSPG4 BsAb [93]: To improve antibody-therapy efficacy in patients with advanced melanoma, the authors developed a BsAb, PD-L1xCSPG4, to selectively reactivate T cells by directing PD-1/PD-L1 disrupting activity to chondroitin sulfate proteoglycan 4 (CSPG4)-expressing tumor cells. A flow cytometry-based assay was employed to evaluate the binding activities of the investigational BsAb. Wild-type ectopically hPD-L1-expressing CHO cells (CHO.PD-L1) were incubated with test antibodies, labeled with a fluorescent secondary antibody, and analyzed by flow cytometry. Dose-dependent binding specific to CHO.PD-L1 cells was observed for the BsAb. These binding activities were replicated in several representative cancer cells endogenously expressing both CSPG4 or PD-L1. In addition, a flow cytometry-based competitive binding assay was used to assess the overall binding strength (avidity) of PD-L1xCSPG4 BsAb to CSPG4+/PD-L1+ cancer cells. BsAb binding was strongly inhibited in the presence of competing parental anti-CSPG4 mAb and only weakly inhibited in the presence of competing PD-L1-blocking mAb. These experiments demonstrate that PD-L1xCSPG4 binds to both PD-L1 and CSPG4 and that the strength of the interaction between the BsAb and CSPG4+/PD-L1+ cancer cells is primarily dominated by binding to CSGA4. To further show that the enhanced binding of the BsAb to CSPG4+/PD-L1+ cells is driven by avidity, cells were pre-incubated with a fluorescent anti-PD-L1 mAb, before being exposed to the test BsAb and a control BsAb, capable of binding PD-L1 but not CSPG4. The EC_50_ of PD-L1xCSPG4 for displacing the probe was substantially lower compared to the control BsAb. Performing the experiment in the presence of an anti-GSPG4 mAb increased the EC_50_ of the PD-L1xCSPG4 BsAb to a level similar to the control BsAb. Together these flow cytometry-based binding assays demonstrated that the PDL1xCSPG4 BsAb has enhanced selectivity for CSPG4+/PD-L1+ cancer cells driven by avidity binding.
- Case Study: Cell-based reporter assay to measure cell surface binding of PDL1xCSPG4 BsAb [93]: The authors further evaluated the role of CSPG4 in mediating the PD-L1-blocking capacity of the PDL1xCSPG4 BsAb using a PD-1/PD-L1 blockade reporter bioassay. The assay relies on co-culturing of Jurkat.PD-1-NFAT-luc reporter T cells (Jurkat cells engineered to express luciferase under the control of a NFAT response element and PD-1) and CHO.PDL1/CD3 cells (CHO cells engineered to express PD-L1 and a membrane-linked agonistic anti-CD3 antibody). Upon successful interaction of PD-1 and PD-L1 between the two cell types, TCR signaling and downstream NFAT-mediated luciferase activity in the Jurkat cells is inhibited. In contrast, interrupting the PD-1/PD-L1 interaction leads to NFAT-mediated luciferase activity. Addition of the PDL1xCSPG4 BsAb to the co-culture disrupted the PD-1/PD-L1 interaction between the two cell types in a dose-dependent manner, as measured by luminescence detection. Next, they tested the role of CSPG4 mAb in PD-1/PD-L1 blocking capacity of PDL1xCSPG4 BsAb by replacing the CHO.PD-L1/CD3 cells with a CSPG4+/PD-L1+ cancer cell line (the CD3 stimulation of T cells was achieved by pre-treating the cells with BIS1; an EpCAM-directed CD3-agonistic bsAb). Stimulated reporter T cells were co-cultured with the double-positive cells in the presence of PDL1xCSPG4 BsAb or controls, with and without anti-CSPG4 mAb. The ability of the PDL1xCSPG4 BsAb to block PD-1/PD-L1 interaction was reduced in the presence of anti-CSPG4 mAb. These findings suggest that the BsAb’s PD-1/PD-L1-disrupting activity will be enhanced against CSPG4+/PD-L1+ cells compared to CSPG4-/PD-L1+ cells.

### 3.3. BsAb Bioassay: Potency Assays

In particular, the strategy of using a potency assay for BsAbs is challenging due to its complicated MoA with two target bindings, and it should be tailored to be MoA-reflective while meeting QC and regulatory expectations to be robust and sensitive methods to detect any structural changes in stability. One interesting question with respect to the BsAb potency assay is if two assays are needed for each target binding or if one potency assay would suffice. Depending on its MoA, either one or two potency assays would be suitable, but it is preferred to have one potency assay to measure synergistic biological effects of two target bindings or a dual read-out of the binding assays in a single assay.

A single assay that can fully capture the bioactivity of the therapeutic molecule is advantageous from both a cost/labor perspective and from a control perspective—synergistic effects resulting from dual antigen binding may be missed if data from multiple assays measuring discrete events are used. However, in order to show the assay is suitably MoA-reflective, the key events in the MoA must be relatively well understood, and characterization assays designed to measure each event (e.g., binding to either antigen, receptor activation, etc.) are needed. The two case studies below describe the development and justification of single QC potency assays to measure changes in bioactivity for (1) a TDB and (2) a DAF that inhibits ligand binding to two distinct cell surface receptors.

- Case Study: Reporter gene T cell activation assay to measure potency of CD3e-binding TDB [96]: The authors developed a reporter gene potency assay that measures T-cell activation in the presence of a CD3e-binding TDB, using Jurkat T-cells engineered to express luciferase under the control of a T-cell activation-sensitive transcriptional response element. The assay was shown to be quantitative and stability indicating. Additionally, it is robust and relatively fast/easy to perform compared to a traditional cell-based cell killing assay, such as a Cr51 release or dye release assay, making it more amenable to a QC testing environment. The MoA of the TDB is complex as it consists of multiple factors—concurrent antigen binding, T-cell activation, and target-cell depletion. In order to show that T-cell activation in an engineered context is a suitable surrogate measure of the TDB’s overall bioactivity, the authors generated a characterization data package consisting of data from individual antigen binding assays (cell-based ELISA to measure binding to the target receptor, SPR to measure binding to CD3) and a flow cytometry-based cell killing assay that used human peripheral blood mononuclear cells as a source of cytolytic T-cells. By using the data generated from the characterization assays, the authors were able to show that changes in potency detected by the reporter gene assay agreed well with changes in affinity for either antigen and cell killing activity. The characterization results support the assertion that the reporter gene assay is sufficiently MoA-reflective to serve as a single potency assay on the control system without the need for additional assays. The authors’ overall strategy can be applied to justify potency for other TDBs with similar MoAs.
- Case Study: Cell-based potency assay to measure biological activity of HER3/EGFR DAF BsAb [69]: In order to measure the activity of a DAF molecule designed to simultaneously inhibit HER3 and EGFR, the authors developed a cell-based potency assay that measures cell proliferation using a cell-permeable redox dye, in which the fluorescence signal is proportional to the number of viable cells. This method was selected based on the molecule’s proposed MoA, which is characterized by blocking ligand binding to each receptor, prevention of receptor dimerization (hetero- and homo-), and inhibition of cell proliferation. A cell line that naturally expresses both receptors and their cognate ligands was selected in order to enable monitoring of the effects of inhibiting both receptors. As in the case study described above, the author’s generated a characterization data package using the potency assay and individual ELISA binding assays for HER3 and EGFR to show that the single potency assay is reflective of the DAF’s overall bioactivity. The fact that the potency assay was demonstrated to be sensitive to changes in affinity for either target and sensitive to inhibition of both receptors, with inhibition of both HER3 and EGFR by the DAF producing the most potent anti-proliferative activity, provides a strong justification that the potency assay sufficiently captures the molecule’s MoA. This, combined with the potency assay’s quantitative ability and stability indicating properties, provides a persuasive argument that it is suitable as a single control system assay for monitoring the impact of product quality on bioactivity.

### 3.4. BsAb Bioassay: Effector Function Assays

Some BsAbs target cell surface proteins or receptors with the intent of enhancing effector function. One arm often targets a tumor-associated antigen while the other targets an immune system-evading surface protein (such as CD47 or CD55/59), increasing susceptibility of the tumor cell to lysis by complement or NK cells, or phagocytosis by macrophages.

Other BsAbs have a primary MoA that does not involve effector function (e.g., TDB, or receptor blocker) but have an effector-competent Fc domain and can also exert cell killing activity through effector function. Depending on the MoA and other molecule-specific factors, effector function can be associated with unfavorable safety events, and so, effector-silenced Fc domains are preferred [110,111]. In other cases effector function enhances a molecule’s activity [54,57].

- Case Study: ADCC reporter assay and competitive ADCP assay to measure enhanced Fc-mediated effector function of GPC3/CD47 BsAb [92]: As mediated by the Fc domain function of therapeutic antibodies, ADCC and ADCP assays are among the appropriate ones to assess the enhanced Fc-mediated effector functions of investigational BsAbs. The authors employed a cell-based reporter system to evaluate the ability of BsAb in inducing ADCC against dual-antigen-expressing Raji-GPC3^H^ cells. In this assay format, engineered Jurkat T lymphocyte cells were used as effector cells. Target cells, including wild-type Raji and Raji-GPC3^H^ cells, were incubated with each mAb and BsAb test antibodies and effector cells. A luminescent substrate was used to measure the luciferase activity at the end of co-incubation that corresponds to the extent of the effector activities. This bioassay revealed that GPC3/CD47 BsAb could induce ADCC against dual-antigen-expressing Raji-GPC3^H^ cells in a dose-dependent manner and to a greater extent compared to the wild-type Raji cells. The ability of BsAb to induce ADCP in vitro was also evaluated upon co-incubation of Raji-GPC3^H^ cells with macrophages. In this assay setup, the effector cells [mouse hSIRPa expressing bone marrow-derived macrophages (BMDMs) harvested from humanized mouse bone marrow] were Alexa Fluor647-labeled and incubated with target cells (Raji or Raji-GPC3^H^ cells) stained with a fluorescent proliferation dye and each antibody. Effector:target cell mixture was then evaluated for ADCP where the phagocytosis of fluorescent-labeled target cells by labeled BMDMs was recorded using a confocal microscope (unphagocytosed cells were washed away prior to microscopy). In this bioassay format, using fluorescence microscopy and quantification of phagocytosis, the authors showed a preferential phagocytosis of dual-antigen-expressing Raji-GPC3^H^ cells specifically in the presence of GPC3/CD47 BsAb.
- Case Study: Use of a CDC assay to assess activity of a complement-regulator neutralizing BsAbs directed against CD20 and CD55/CD59 [75]: The authors designed BsAbs to increase complement-mediated killing of CD20-expressing B cells. By simultaneous targeting CD20 and CD55/CD59, the BsAbs are able to neutralize the C-regulating proteins on B cells, leading to more efficient killing by CDC. Various CD20-expressing cells were treated with the BsAbs, followed by incubation with human sera (source of complement). Following an incubation period, cells were assessed for viability using MTT (a dye that is reduced to form a purple dye in the presence of metabolically active cells). Cell killing was enhanced by treatment with the BsAbs compared to treatment with an effector-competent aCD20 mAb. Additionally, cell killing levels remained consistent in the presence of CD20-bystander cells expressing CD55 and CD59, suggesting that the BsAbs are selectively killing CD20+ B cells. Flow cytometry-based binding assays were used to confirm binding to cells expressing CD20, CD55, and CD59.

### 3.5. BsAb Bioassay: Impurities Assays

Impurities assays for BsAbs are often physicochemical assays such as size-exclusion chromatography (to measure aggregates and fragments), imaging capillary isoelectric focusing/ion exchange chromatography (to measure charge variants), and mass spectrometry (to sensitively identify and/or quantify post-translational modifications and other trace variants) [112], which are commonly used to characterize impurities for conventional mAbs. However, the unique structure of BsAbs can produce unique product variants with impacts to safety and/or bioactivity that are not fully addressed by physicochemical assays. The nature/activity of such impurities is rooted in the structure of the molecule, its production process, and MoA. In order to illustrate this point a case study describing the development of a bioassay to measure T-cell activating impurities for a TDB is described below.

- Case Study: Luciferase reporter T cell activation assay to measure functional effects of impurities on CD3e-targeting TDB [95]: A CD3e-targeting TDB produced by knobs-into-holes technology and assembled in vitro contains a number of product-related impurities with the potential to activate T-cells in the absence of target cells. For example, aggregates and aCD3 homodimer, which result from the mispairing of aCD3 half antibody fragments during production, are characterized by multivalent binding to CD3 and can crosslink the TCR resulting in activation. These impurities are a safety concern because T-cell activation is linked to adverse events such as cytokine release syndrome. While aggregates and aCD3 HD can be measured using analytical methods, a bioassay is needed to assess their biological impact, such as target-independent T-cell activation. To address this need, the authors developed a reporter gene assay that measures T-cell activation in the absence of target cells using Jurkat T-cells engineered to express luciferase when activated. T-cell activation of product-related impurities present in the TDB formulation was quantified relative to T-cell activation by aCD3 HD standard. Using this assay, the authors were able to characterize the T-cell activating activities of aggregates and other product-related impurities, in order to get an idea of their potential impacts to safety and inform on the overall control strategy. Additionally, because the assay is a “catchall” assay that measures the combined T-cell activating activity of product-related impurities that may be present in a given sample, the method is able to provide reassurance that combinations of impurities are not leading to unexpected T-cell activation. Such combination effects would not be identified using physicochemical methods alone.

## 4. Conclusions

BsAbs represent a highly promising and emerging therapeutic area. Due to structural and biological differences from monospecific Abs, development of a bioassay strategy for the BsAb poses unique challenges and considerations. We reviewed currently available bioanalytical technological platforms, bioassays, and relevant case studies for BsAbs to provide insight into designing a BsAb release and characterization strategy. Understanding and developing good bioassays are critical for the overall control strategy of BsAbs to measure biological activities, and they will continue to evolve for both BsAb molecules and analytical technologies available.

## Figures and Tables

**Table 1:** List of bioassays for bispecific molecules.

Bioassay	Method Principle	Examples
Bridging ELISA	To assess the ability of each arm of the BsAbs to bind two antigens simultaneously. The assay follows a sequential capture method, where antibody is allowed to bind the first coated antigen, followed by a wash step and addition of a biotinylated version of the second antigen. The bound biotinylated antigen can be detected using HRP-labeled streptavidin and luminescent substrate.	tetra-VH IgG bispecific tetravalent [86]
Sandwich ELISA	To assess binding specificity, including dual-specificity detection, of BsAbs. The assay format consists of antigen incubation with an immobilized capture antibody in the plate, followed by wash step to remove non-bound components. The antibody-antigen complex is then detected using a labeled antibody.	IgE receptor signaling blocking BsAb, FcεRI/FcγRIIb cross-link [87]
Bridging SPR	To measure the binding affinities of antibodies to their respective antigens. The assay follows Biacore™ SPR-based format, where two sequential binding events to a ligand immobilized on a chip and surface regeneration is used to measure a bridging signal and, as a result, the simultaneous binding of the assay to both antigens.	Ang-2/VEGF BsAb [88]
Dual-Binding SPR	A solution binding SPR-based assay for individual assessment of both targets in solution without the need for immobilization and regeneration of the target.	anti-VEGFA-121/Ang2 BsAb [89]
Direct Cell Killing	To evaluate cell killing potential by co-culturing the target and effector cells in the presence or absence of BsAb. Assay readout can be accomplished through luciferase reporter system or by flow cytometry-based methods to measure percent apoptotic cells or percent cytolysis of pre-labeled target cells by proliferation dye dilution analysis. Additional assays include labeling target cells with ^51^Cr or measuring the presence of extracellular LDH, where the release of the label or LDH by lysed target cells is used as surrogate for cell-killing activity [90,91].	CD47 blocking BsAb specifically targeted to GPC3 expressing target cells [92]) PD-L1 blocking BsAb specifically directed to CSPG4-expressing target cells [93] CD3-bispecific (anti-HER2/CD3) TDB [94]
T Cell Activation	To assess BsAb effects on T-cell activation and proliferation potential. Assay readout mainly includes luciferase bioluminescence reporter signal using Jurkat T cells engineered with an NFAT-response-element driving luciferase expression. Depending on the MoA of the molecule, T-cell activation can be triggered either only by T cells expressing relevant receptors or in the presence of antigen-presenting target cells. In addition, activation and proliferation of T cells can be evaluated using an in-vitro mixed lymphocyte reaction followed by flow cytometry (proliferation dye dilution analysis or measurement of T-cell activation markers) and ELISA (for IFN-g and granzyme B secretion measurements).	CD3e-targeting TDB [95] PD-L1 blocking BsAb specifically directed to CSPG4-expressing target cells [93] CD3-bispecific (anti-HER2/CD3) TDB [94]

BsAbs: bispecific antibodies; ELISA: enzyme-linked immunosorbent assay; HRP: horseradish peroxidase; IFN-g: interferon gamma; LDH: lactate dehydrogenase; MoA: mechanism of action; NFAT: nuclear factor of activated T cells; SPR: surface-plasmon resonance; VH: variable heavy domain; Ang-2: angiopoietin-2; VEGF: vascular endothelial growth factor; PD-L1: programmed death-ligand 1; CSPG: chondroitin sulfate proteoglycan.

**Table 2:** BsAb categories and potential bioassays applicable: Summary of case studies.

	Type 1 Cell Bridging	Type 2 Receptor Blocking or Activating (Cis/Trans)	Type 3 Cofactor Mimicking	Type 4 Spatial Targeting (“Homing” BsAbs)
Binding	ELISA (binding to single target) [96], SPR (binding to single target) [96]	ELISA (binding to either target), SPR (affinity for either target) [50], bridging ELISA (dual target recognition) [50], bridging SPR [92], ELISA (ligand blocking) [74], SPR (stoichiometry of binding) [74]	SPR (characterize affinity for FIX, FIXa, FX, FXa) [61,62], ELISA (confirm specificity for FIX and FX) [62]	BLI (measure affinity of each arm, and support 1:1 binding) [68], Competition ELISA [63]
Bioactivity (Major MoA)	Reporter gene effector cell activation assay [96], direct cell killing assay [21,22,42]	Cell Proliferation [50,69,76], Apoptosis [73], cytokine neutralization [56]	Enzymatic assays (FXa activity) [61]	Viral Inactivation [68], TR-FRET AB assay [63]
Functional (other supporting MoA as characterization)	Cell depletion by flow cytometery [21,96], Cytokine release [42], cell surface marker expression (per MoA) [22,42,45,96]	Tyrosine phosphorylation [74], inhibition of antibody production/secretion [50], Calcium flux assay [50]	Thrombin generation assay [61,62]	Fluorescence microscopy to assess subcellular localization [63,65,68], transcytosis assay [65]
Effector Function	ADCC [97], ADCP [97]	CDC assay [75], ADCC/ADCP bioassay [92], binding to FcgRs [92]	NA	NA
Impurity Bioassay	T-cell activating impurities [95]	NA	NA	NA

BLI: biolayer interferometry; TR-FRET: time-resolved fluorescence resonance energy transfer.

## Data Availability

No new data were created or analyzed in this study. Data sharing is not applicable to this article.
